# Supplementary material for: ERp18 regulates activation of ATF6α during unfolded protein response
Source: EMBO J. 2019 Jun 17;38(15):e100990. doi: 10.15252/embj.2018100990 (PMC6670016; doi:10.15252/embj.2018100990)
Supplement: Supplementary file 1 — Expanded View Figures PDF [file EMBJ-38-e100990-s001.pdf]

Expanded View Figures

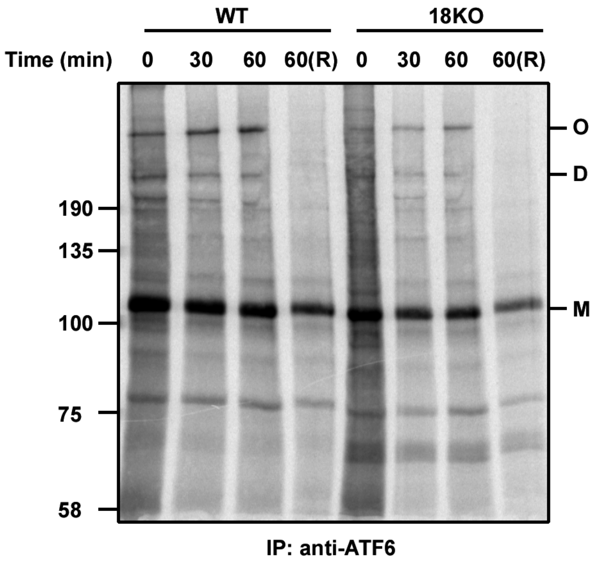

Figure EV1. ERp18 KO does not effect ATF6alpha disulfide formation.
ATF6alpha was immunoisolated using mouse anti-ATF6. Samples were separated by SDS-PAGE under non-reducing condition apart from lane 4 and 8 where they were reduced prior to separation.

© 2019 The Authors
