# Supplementary material for: ERp18 regulates activation of ATF6α during unfolded protein response
Source: EMBO J. 2019 Jun 17;38(15):e100990. doi: 10.15252/embj.2018100990 (PMC6670016; doi:10.15252/embj.2018100990)
Supplement: Supplementary file 2 — Table EV1 [file EMBJ-38-e100990-s002.docx]

**Table EV1**

**Mutagenesis Primers**

ATF6_FW_NheI AATTGCTAGCGCCACCATGGCTTCTAGCTATCC

ATF6_RV_V5-1 GAATCAAGACCAAGAAGAGGATTAGGAATAGGTTTACCTTGT

AATGACTCAGGG

ATF6_RV_NotI_V5-2 GTACTTGCGGCCGCTTAAGTAGAATCAAGACCAAGAAGAG

ATF6_C467A_FW CCACCTCCTGCTCAGCCCCTA

ATF6_C467A_RV TAGGGGCTGAGCAGGAGGTGG

ATF6_C618A_FW CAGATTGACGCTCAGGTGATG

ATF6_C618A_RV CATCACCTGAGCGTCAATCTG

ATF6_D564G_FW CATCCGCAGAAGGGGAGGCACATTTTATGTTGTG

ATF6_D564G_RV CACAACATAAAATGTGCCTCCCCTTCTGCGGATG

**CRISPR guides**

Guide_14 ttgttGAAACTGAAGCCCAGCAAAC

Guide_8 caccGATGGACATAATGGGCT

Guide_10 caccGGACATAATGGGCTTGGAA

**qPCR Primers**

GRP78 (BiP): 5’-CATGGTTCTCACTAAAATGAAAG-3’(FW)

5’-GCTGGTACAGTAACAACTG-3’(RV)

XBP1s: 5’-GGAGTTAAGACAGCGCTTGG-3’(FW)

5’-CCTGCACCTGCTGCG-3’(RV)

GAPDH: 5’-TGGAAGATGGTGATGGGATT-3’(FW)

5’-AGCCACATCGCTCAGACAC-3’(RV)

ATF4: 5′ GTCCCTCCAACAACAGCAAG3′(FW)

5′ AGGTCATC TGGCATGGTTTC3′(RV)

GRP94: 5' -GGAGAGTCGTGAAGCAGTTGAG-3'(FW)

5'- CCACCAAAGCACACGGAGATTC-3'(RV)

ATF6: 5' -CAGACAGTACCAACGCTTATGCC -3'(FW)

5' -GCAGAACTCCAGGTGCTTGAAG -3'(RV)
