# Supplementary figures and images for: ERp18 regulates activation of ATF6α during unfolded protein response
Source: EMBO J. 2019 Jun 17;38(15):e100990. doi: 10.15252/embj.2018100990 (PMC6670016; doi:10.15252/embj.2018100990)

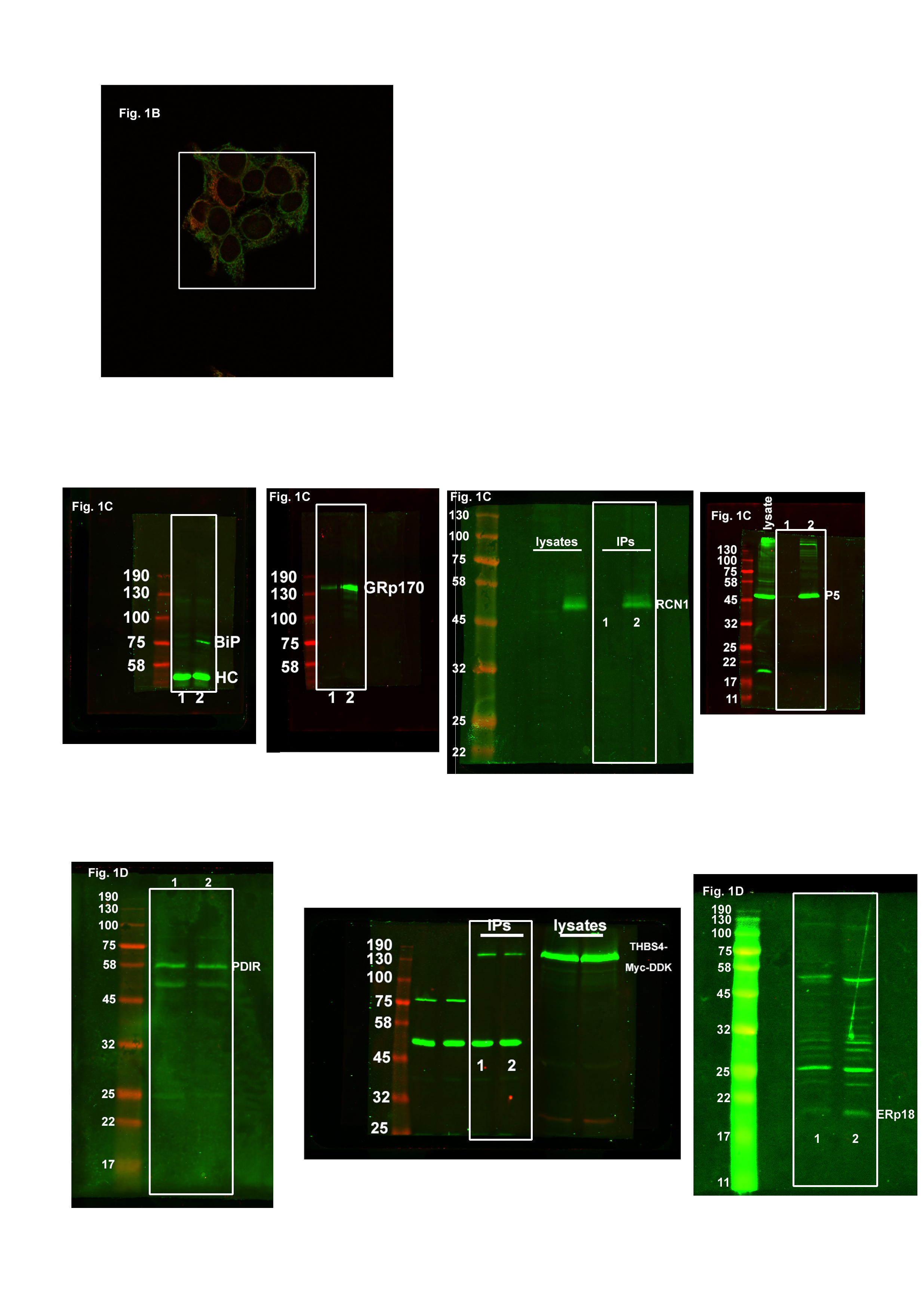

Supplement: Supplementary file 4 — Source Data for Figure 1 [file EMBJ-38-e100990-s003.jpg]

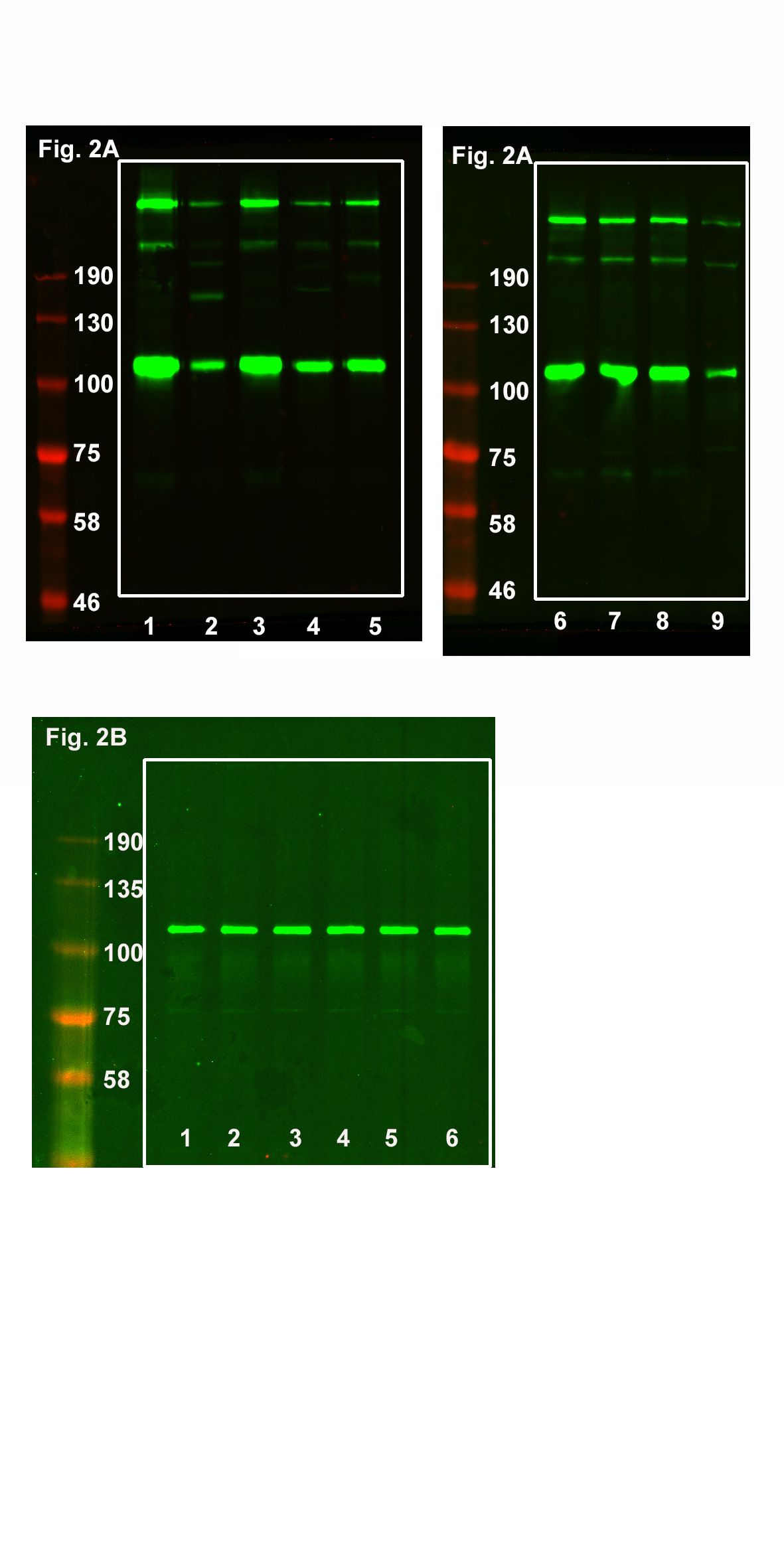

Supplement: Supplementary file 5 — Source Data for Figure 2 [file EMBJ-38-e100990-s004.jpg]

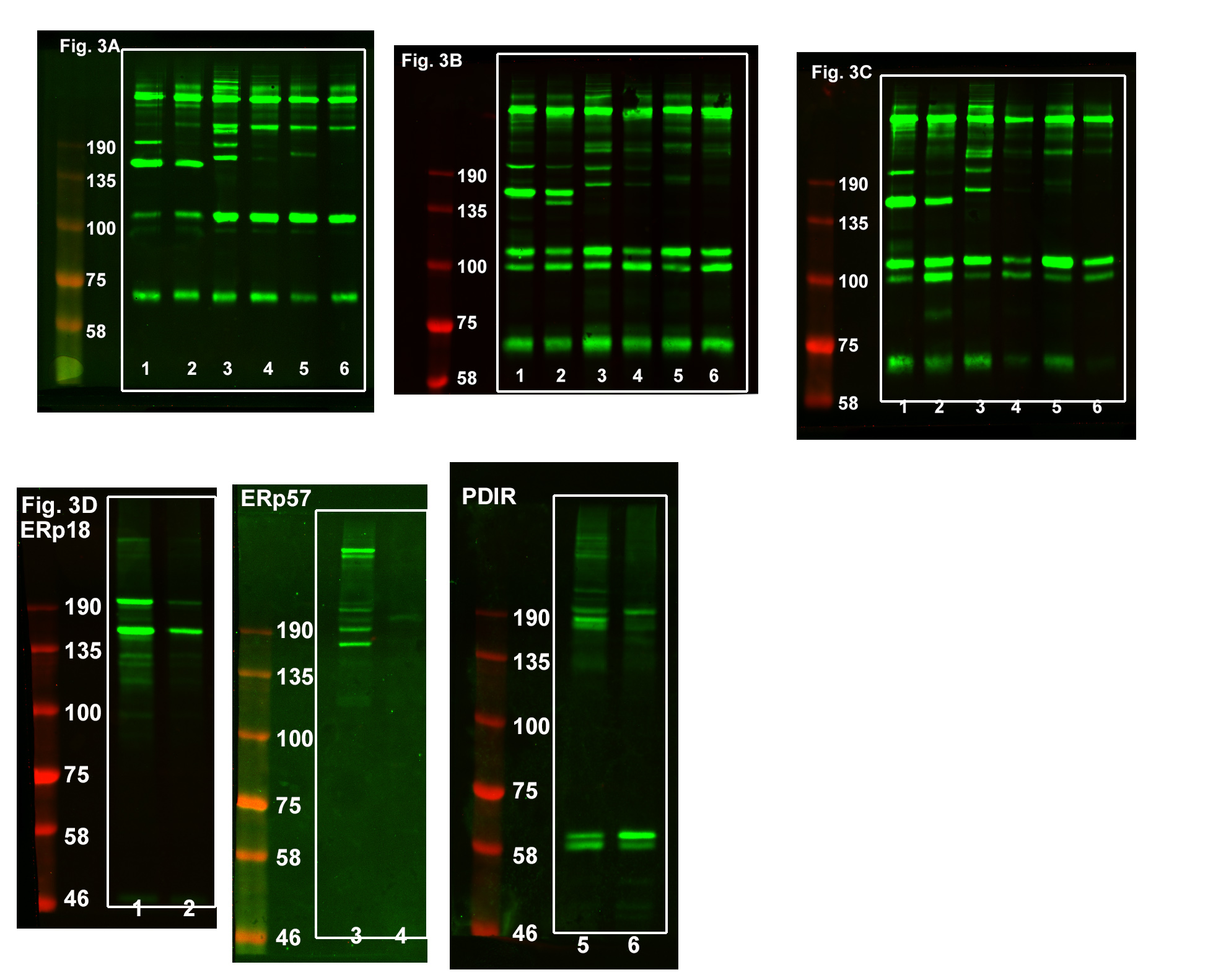

Supplement: Supplementary file 6 — Source Data for Figure 3 [file EMBJ-38-e100990-s005.jpg]

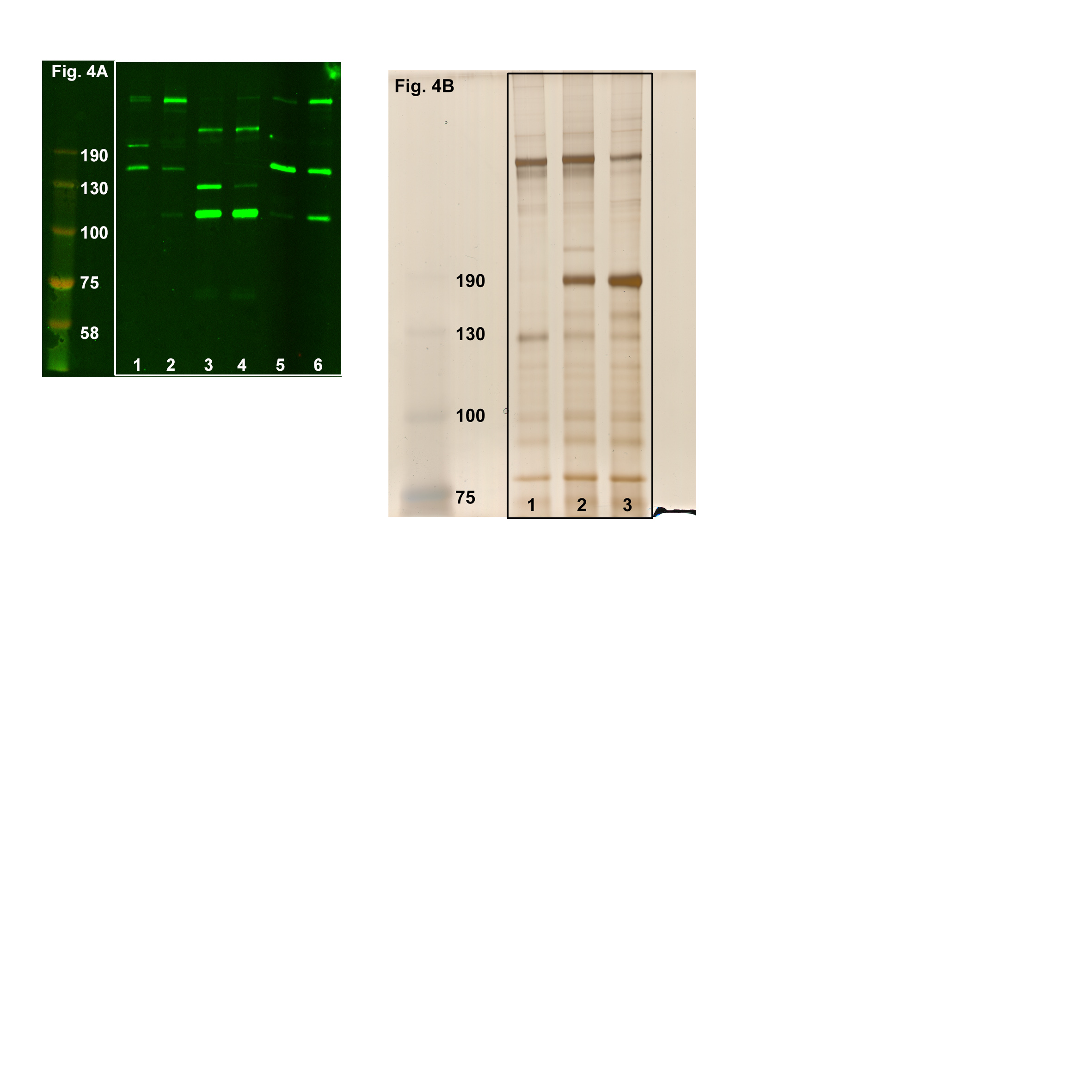

Supplement: Supplementary file 7 — Source Data for Figure 4 [file EMBJ-38-e100990-s006.jpg]

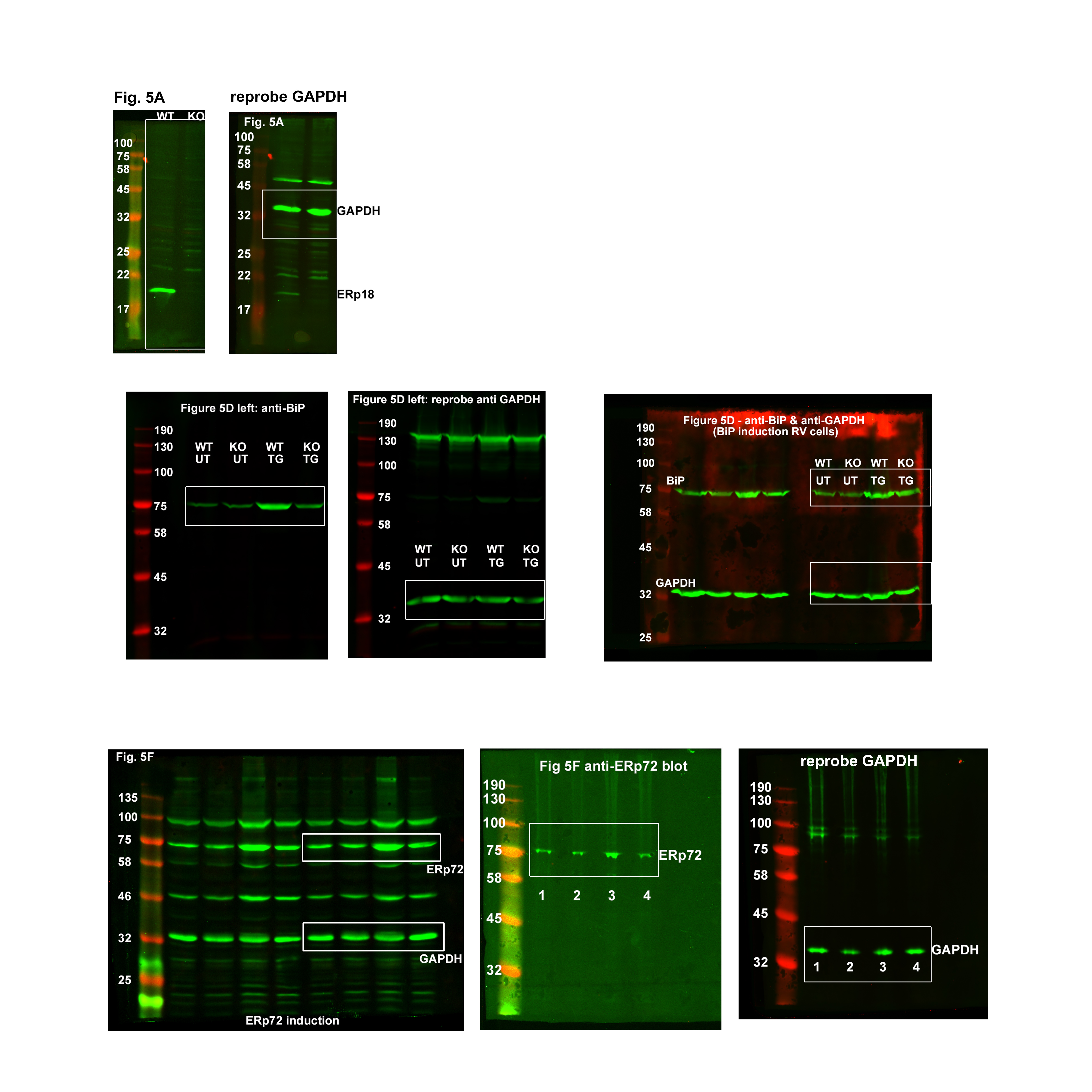

Supplement: Supplementary file 8 — Source Data for Figure 5 [file EMBJ-38-e100990-s007.jpg]

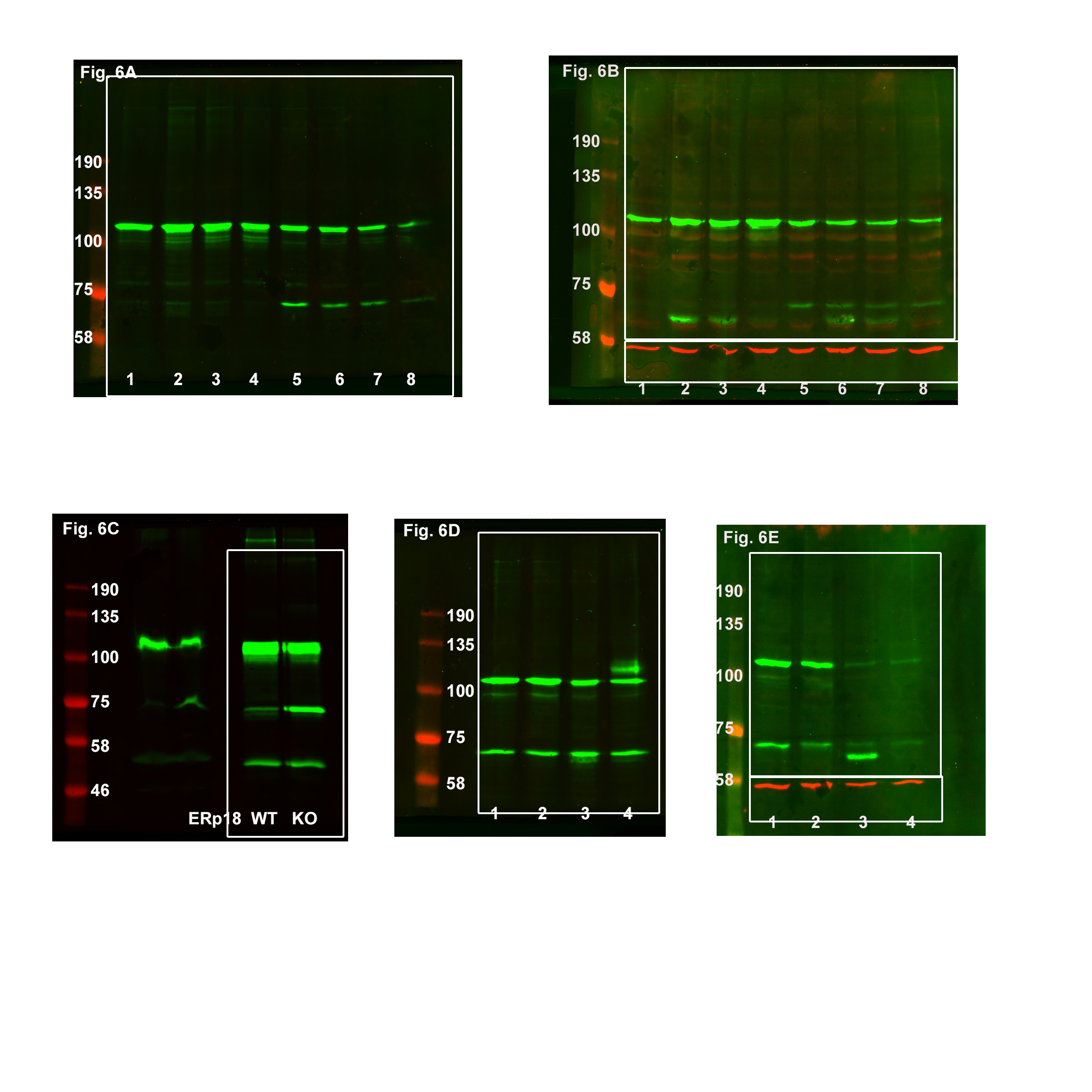

Supplement: Supplementary file 9 — Source Data for Figure 6 [file EMBJ-38-e100990-s008.jpg]

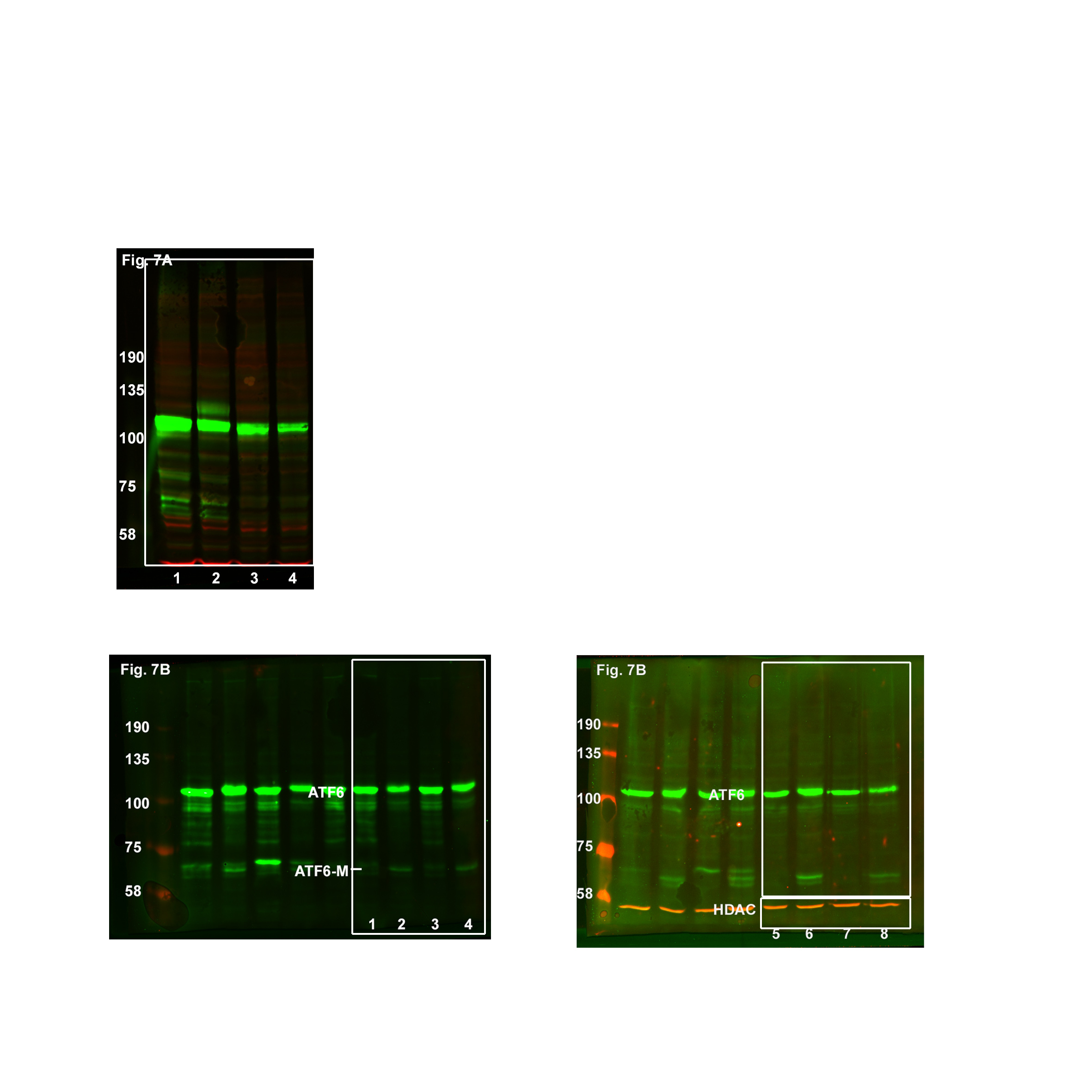

Supplement: Supplementary file 10 — Source Data for Figure 7 [file EMBJ-38-e100990-s009.jpg]

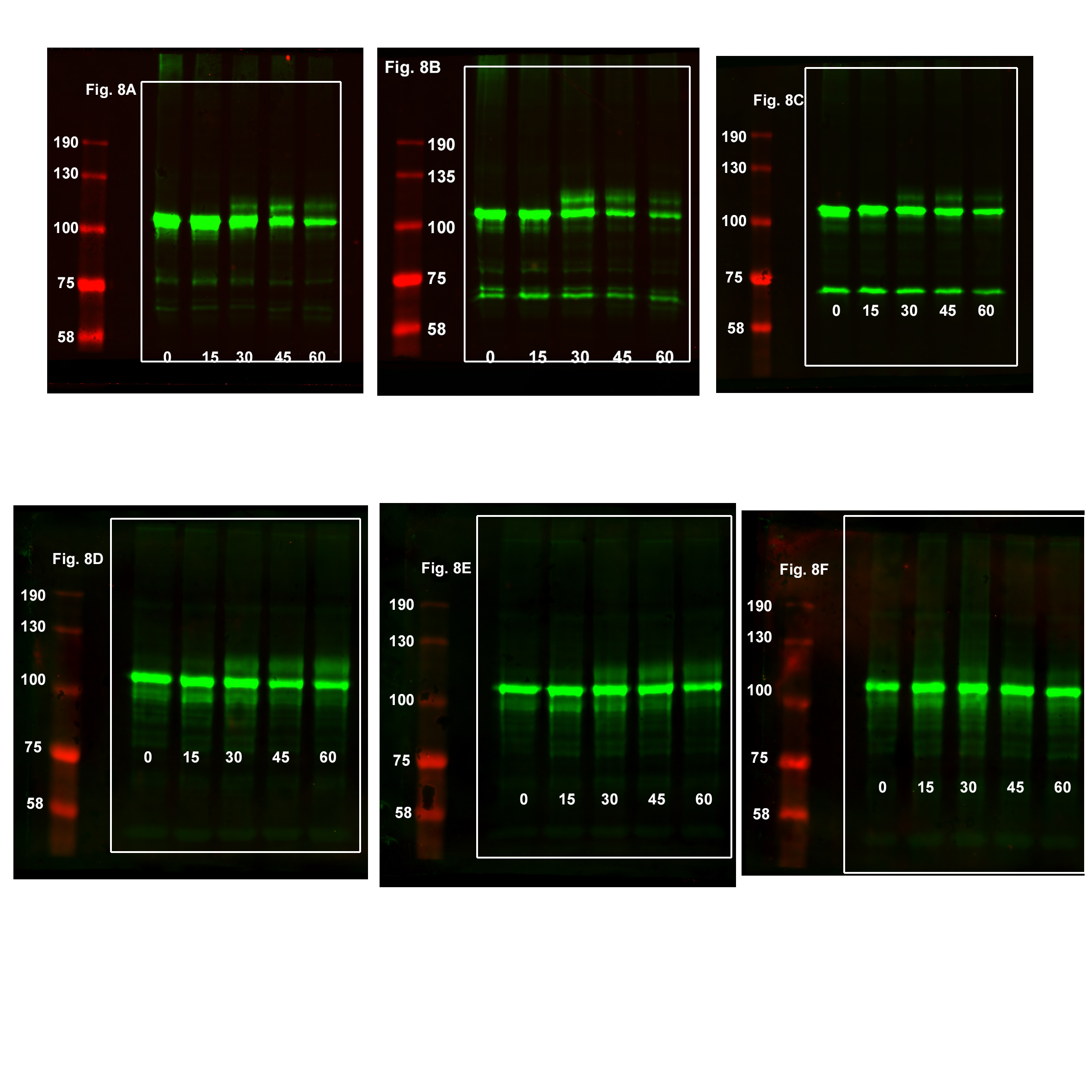

Supplement: Supplementary file 11 — Source Data for Figure 8 [file EMBJ-38-e100990-s010.jpg]
